# Comparison of Phenotypic and Genotypic Antimicrobial Resistances in Bovine *Clostridioides difficile* Isolates

**DOI:** 10.3390/antibiotics15050495

**Published:** 2026-05-14

**Authors:** Ines Unger, Mostafa Y. Abdel-Glil, Jutta Lox, Gernot Schmoock, Heinrich Neubauer, Stefan Schwarz, Christian Seyboldt

**Affiliations:** 1 Institute of Bacterial Infections and Zoonoses, Friedrich-Loeffler-Institut, Federal Research Institute for Animal Health, Naumburger Straße 96a, 07743 Jena, Germanygernot.schmoock@fli.de (G.S.); heinrich.neubauer@fli.de (H.N.); christian.seyboldt@fli.de (C.S.); 2 Institute of Microbiology and Epizootics, Centre for Infection Medicine, School of Veterinary Medicine, Freie Universität Berlin, 14163 Berlin, Germany; stefan.schwarz@fu-berlin.de; 3 Veterinary Centre for Resistance Research (TZR), School of Veterinary Medicine, Freie Universität Berlin, 14163 Berlin, Germany

**Keywords:** antimicrobial resistance determinants, agar dilution, *Clostridium difficile*, cattle, WGS

## Abstract

**Background/Objectives**: Antimicrobial agents play an important role in the pathogenesis and treatment of *Clostridioides* (*C*.) *difficile* infections. *C. difficile* isolates have shown different genotypic and phenotypic resistance patterns and could serve as antimicrobial resistance reservoirs. **Methods**: To gain insight into accordance and potential disagreements between genotypic and phenotypic antimicrobial resistances in *C. difficile*, we compared the genotypic and phenotypic resistance patterns of 108 bovine *C. difficile* isolates collected in Germany between 2010 and 2012. These isolates represent a collection of different ribotypes (RT) and originated from different husbandries in Germany. Whole genome sequencing of all isolates was performed with Illumina^®^ Miseq™, and sequences were screened for antimicrobial resistance determinants. For phenotypic antimicrobial susceptibility testing, the agar dilution procedure according to the CLSI document M11 was used. Minimal inhibitory concentration values were determined for penicillin, meropenem, tetracycline, moxifloxacin, vancomycin, metronidazole, erythromycin and clindamycin. **Results**: Various phenotypic and genotypic antimicrobial resistances were found in the isolates examined that belonged to different ribotype/sequence type (ST) lineages, even if these originated from the same source and geographical region (bovine isolates from Germany). Agreement between phenotypic and genotypic resistance was seen for most antimicrobial agents tested. A total of 92% (83/90) of the investigated ST11 isolates showed phenotypic resistance or were classified as non-wild type to at least one of the antimicrobials tetracycline, moxifloxacin, erythromycin and clindamycin. **Conclusions**: The results of this comparison contribute to a better understanding of antimicrobial resistance in *C. difficile* by relating phenotypic susceptibility patterns to genomic resistance determinants.

## 1. Introduction

Nowadays, infections with antimicrobial-resistant pathogens are a major problem. An estimated 5 million people worldwide died in 2019 in connection with infections by antimicrobial-resistant bacteria [1]. The U.S. Centers for Disease Control and Prevention classified *Clostridioides* (*C*.) *difficile* as one of the most important pathogens in terms of the threat of antimicrobial resistance in a 2019 report [2]. *C. difficile* is a Gram-positive, spore-forming, anaerobic and presumably zoonotic bacterium [3,4,5]. It is ubiquitous in the environment and in the intestinal tract of various macroorganisms and can cause severe diseases in humans and animals. Symptoms range from mild diarrhea to death, but asymptomatic carriage is also possible [5,6,7].

The epidemiology of this pathogen is closely linked to antibiotics. The bacterium was first described in 1935 [8]. After an increase in colitis cases following treatment with the broad-spectrum antibiotic clindamycin, *C. difficile* was described in several publications as a potential trigger of this antimicrobial-associated colitis [8,9,10]. With the use of third-generation cephalosporins, the number of *C. difficile* infections rose sharply in the 1980s and 1990s [11]. Shortly thereafter, ribotype (RT) 027, which spread worldwide due to its resistance to fluoroquinolones [12,13], led to a sharp increase in the incidence and mortality of *C. difficile* infections [14].

*C. difficile* strains exhibit a variety of phenotypic and genotypic antimicrobial resistances, allowing *C. difficile* to serve as a reservoir for antimicrobial resistance [15]. Some sequence type (ST)/RT lineages are of particular interest in this regard, e.g., ST11. This lineage, including the main sublineage RT078, is livestock-associated and harbors various antimicrobial resistance genes, primarily against aminoglycosides and tetracyclines [12,16]. However, different resistances can also be found in other RTs [6]. Phenotypic and genotypic resistance do not always coincide, and many resistance mechanisms are not yet fully understood [15].

Data on *C. difficile* antimicrobial resistance (AMR) in cattle are limited. Most isolates from bovine feces are susceptible to metronidazole and vancomycin [17,18,19,20,21,22,23,24,25]; however, metronidazole- or vancomycin-resistant *C. difficile* isolates from bovine feces or carcasses have been reported in Italy, the United States, Turkey, Iran and Ireland [17,24,26,27,28]. The isolate from an Irish bovine carcass even showed resistance to both antimicrobial agents [27]. Vancomycin and metronidazole are among the recommended antimicrobial agents for the treatment of *C. difficile* infections in humans [29,30,31]. Although the prevalence of metronidazole- and/or vancomycin-resistant strains is generally low, their detection is a cause for concern as they could potentially spread to the human population [27]. Resistance to antibiotics commonly used in agricultural settings (e.g., tetracycline) is widespread, and high resistance rates for tetracycline (up to 93.1% after treatment with oxytetracycline) can be detected in isolates from bovine feces [14,18,32,33]. Resistance to macrolides or lincosamides, particularly erythromycin or clindamycin, is common in bovine or bovine farm-associated *C. difficile* isolates [17,18,19,20,21,22,23,24,25,28,33], with minimal inhibitory concentration (MIC) values up to >512 mg/L (erythromycin) and 512 mg/L (clindamycin) [20]. Resistance to the fluoroquinolone moxifloxacin was observed in isolates from bovine feces in Brazil, the United States and Italy [18,22,23]. Ampicillin-resistant isolates have been detected in the United States (bovine feces) and Turkey (bovine carcasses) [24,26]. All isolates investigated in a Turkish study were classified as resistant to ciprofloxacin, whereas resistance rates of 33.3% and 25% to ciprofloxacin were measured in studies from Iran and China (all bovine feces) [19,21,28]. Several antimicrobial resistance determinants have been detected in bovine *C. difficile* isolates, e.g., the aminoglycoside resistance genes *ant*(6)-Ia, *ant*(6)-Ib and *aadE*, the streptothricin resistance gene *sat4*, different tetracycline resistance genes (*tet*(M), *tet*(S), *tet*(44)*, tet*(O), *tet*(W), and *tet*(40)), mutations in *gyrA* and *gyrB* that can cause fluoroquinolone-resistance, genes coding for methyltransferases (*cfr*, *erm*(B)), and the beta-lactamase gene *bla*_CDD_ [15,17,18,32,33,34,35].

The aim of this study was to descriptively compare phenotypic antimicrobial susceptibility profiles with WGS-based detection of AMR determinants across selected antimicrobial classes in 108 bovine *C. difficile* strains isolated in Germany between January 2010 and August 2012 [36]. Although the isolate collection does not necessarily reflect the current situation of circulating *C. difficile* strains in German cattle husbandry, it still provides valuable insight into the AMR characteristics of bovine *C. difficile* isolates. Thus, the results of this study contribute to a better understanding of AMR in this pathogen and expand the available data. To our knowledge, this is the first study combining phenotypic and genotypic AMR characterization of bovine *C. difficile* isolates from Germany.

## 2. Results

A total of 108 *C. difficile* isolates from cattle in Germany (collected between 2010 and 2012) were included in this study to investigate their AMR patterns Detailed information on the source population, sampling framework, and available background data for these isolates has been reported previously [36]. The isolates were characterized using whole genome sequencing data (*n* = 108 isolates) and phenotypic antimicrobial susceptibility testing (AST) by agar dilution (*n* = 106; two strains (both RT014/0) were excluded due to insufficient growth on the control plates). The results for individual isolates can be found in Tables S1 and S2 of the Supplementary Materials.

### 2.1. Beta-Lactams

*C. difficile* is intrinsically resistant to most β-lactam antibiotics [15], the most widely prescribed antimicrobial class in the United States [37]. These antibiotics are strongly associated with *C. difficile* infections in humans [15]. Resistance to β-lactam antibiotics in *C. difficile* is primarily mediated by the production of β-lactamases, which cleave and inactivate the β-lactams. The expression of these enzymes is regulated by associated sensor and repressor genes, including *blaR* and *blaI*, which control the inducible β-lactamase response in this species [15,38].

**Figure 1:**
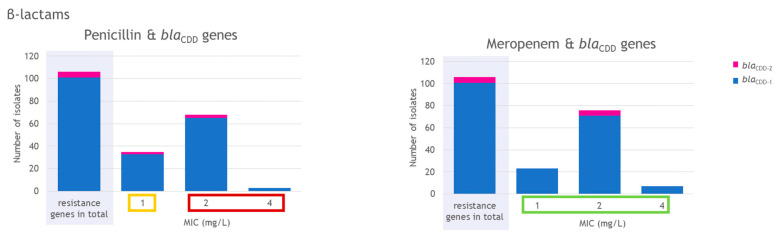
Detected *bla*_CDD_ genes and minimal inhibitory concentration (MIC) values for penicillin and meropenem. Results for RT014/0 isolates are not included in this figure (they were excluded from the phenotypic antimicrobial susceptibility testing (AST) due to insufficient growth on the control plates). MIC values are marked as susceptible (green), intermediate (yellow) or resistant (red) according to CLSI breakpoints [40].

In all isolates, the gene *blaR1* was present, which codes for a beta-lactam sensor/signal transducer. Furthermore, *bla*_AHM_, coding for an AHM family subclass B3-like metallo-beta-lactamase [39], was present in all isolates. Every isolate also harbored a *bla*_CDD_ gene. In 95.4% (103/108) of the isolates, *bla*_CDD-1_ was detected, whereas *bla*_CDD-2_ was detected in the remaining five isolates (Figure 1; Table S3.3). The *bla*_CDD-2_–positive isolates belonged to ribotypes RT003/FLI01 (100%, 1/1), RT010 (100%, 3/3), and RT023/FLI01 (100%, 1/1).

**Figure 2:**
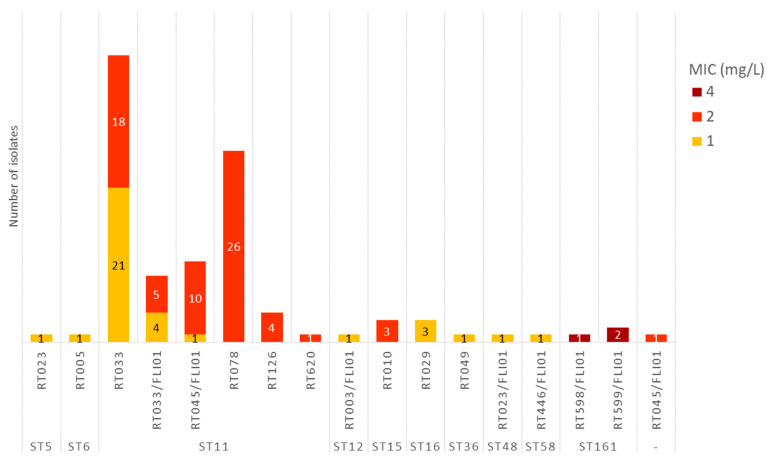
MIC values for penicillin depending on sequence type (ST) and ribotype (RT). Results for RT014/0 isolates are not included in this figure (they were excluded from the phenotypic AST due to insufficient growth on the control plates).

Phenotypic antimicrobial susceptibility testing showed that for penicillin, 33.0% (35/106) of the isolates were classified as intermediate, exhibiting MIC values of 1 mg/L. The majority of isolates (64.2%, 68/106) had a MIC value of 2 mg/L, and 2.8% (3/106) had a MIC value of 4 mg/L and were therefore classified as resistant (Figure 1). The three penicillin-resistant isolates with MIC values of 4 mg/L belonged to ribotypes RT598/FLI01 and RT599/FLI01 (Figure 2).

**Figure 3:**
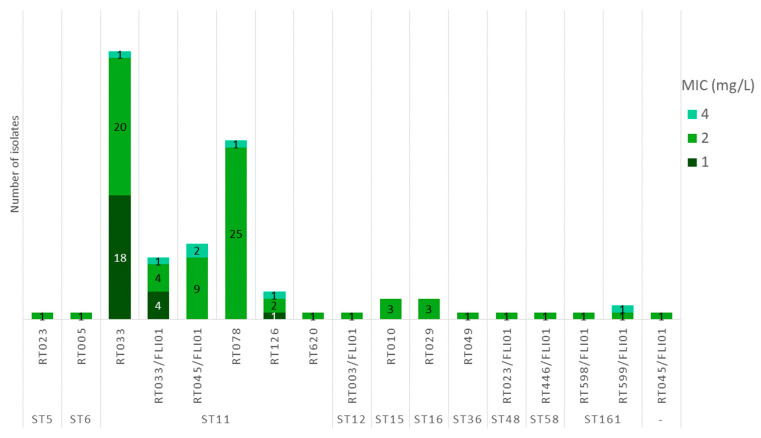
MIC values for meropenem depending on ST and RT. Results for RT014/0 isolates are not included in this figure (they were excluded from the phenotypic AST due to insufficient growth on the control plates).

Meropenem, tested as a representative of the carbapenems, showed full susceptibility across all isolates, with MIC values ranging from 1 to 4 mg/L (Figure 1). According to CLSI document M100, *C. difficile* isolates were classified as meropenem susceptible, intermediate or resistant when they exhibited MIC values of ≤4, 8, or ≥16 mg/L, respectively [40]. The highest MIC value of 4 mg/L was measured for isolates belonging to ST11 (different ribotypes) and RT599/FLI01 (ST161) (Figure 3).

### 2.2. Fluoroquinolones

**Figure 4:**
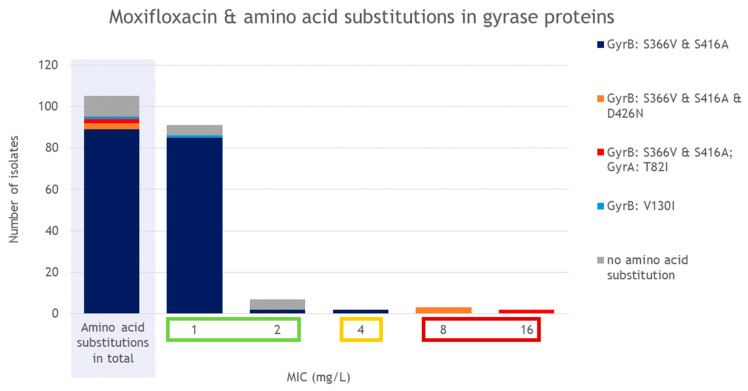
Detected amino acid substitutions in gyrase proteins and MIC values for moxifloxacin. Results for RT014/0 isolates are not included in this figure (they were excluded from the phenotypic AST due to insufficient growth on the control plates). MIC values are marked as susceptible (green), intermediate (yellow) or resistant (red) according to CLSI breakpoints [40].

**Figure 5:**
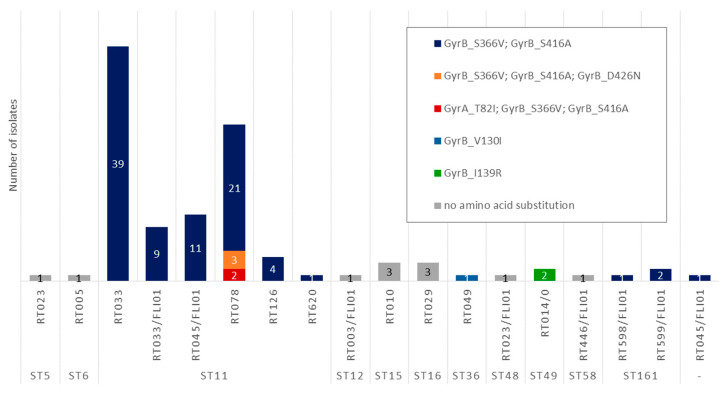
Presence of amino acid substitutions depending on ST and RT.

Fluoroquinolone resistance in *C. difficile* is primarily associated with point mutations in the gyrase genes, *gyrA* and *gyrB*, which alter the quinolone-binding region of DNA gyrase [15]. Most of these mutations that lead to fluoroquinolone resistance are located in the so-called quinolone resistance-determining regions (QRDR: in GyrA located at amino acid positions 25 to 153, and in GyrB at positions 353 to 483) [15]. However, (i) not all amino acid substitutions in the QRDR regions are automatically associated with fluoroquinolone resistance and (ii) mutations outside these regions have also been described in association with fluoroquinolone resistance in *C. difficile* [15,41,42]. In 89.8% (97/108) of the isolates, mutations in the gyrase genes were detected that led to amino acid substitutions (Figure 4). Among the 94 isolates belonging to ribotypes RT033, RT033/FLI01, RT045/FLI01, RT078, RT126, RT598/FLI01, RT599/FLI01, and RT620, the substitutions S366V and S416A were consistently found in GyrB (Figure 5). Of these, three isolates (RT078) carried an additional GyrB substitution (D426N) while two other isolates (RT078) carried an additional substitution (T82I) in GyrA. The substitution I139R in GyrB was present in both RT014/0 isolates, and the substitution V130I was present in the single RT049 isolate (Figure 5).

Phenotypic susceptibility testing to moxifloxacin (Figure 4) showed that five RT078 isolates were classified as resistant. These isolates were the ones that harbored the amino acid substitutions D426N in GyrB (MIC of 8 mg/L) or T82I in GyrA (MIC of 16 mg/L). Most of the isolates were classified as susceptible to moxifloxacin with a MIC of 1 mg/L (85.8%, 91/106) or 2 mg/L (7.5%, 8/106). Two RT033 isolates revealed a MIC of 4 mg/L and were, therefore, classified as intermediate.

### 2.3. Macrolide–Lincosamide–Streptogramin Antimicrobials

Resistance to erythromycin and/or clindamycin is among the most common antimicrobial resistance phenotypes in *C. difficile*. Resistance mechanisms include ribosomal methylation mediated by *erm* genes, efflux pumps, and drug inactivation [43].

Genotypic analysis revealed that the gene *cplR* was detected in 91.7% (99/108) of the isolates. It was absent in nine isolates belonging to RT045/FLI01. This gene codes for a miscellaneous ABC-F subfamily ATP-binding cassette ribosomal protection protein which contributes to intrinsic pleuromutilin, lincosamide and streptogramin A resistance [44].

**Figure 6:**
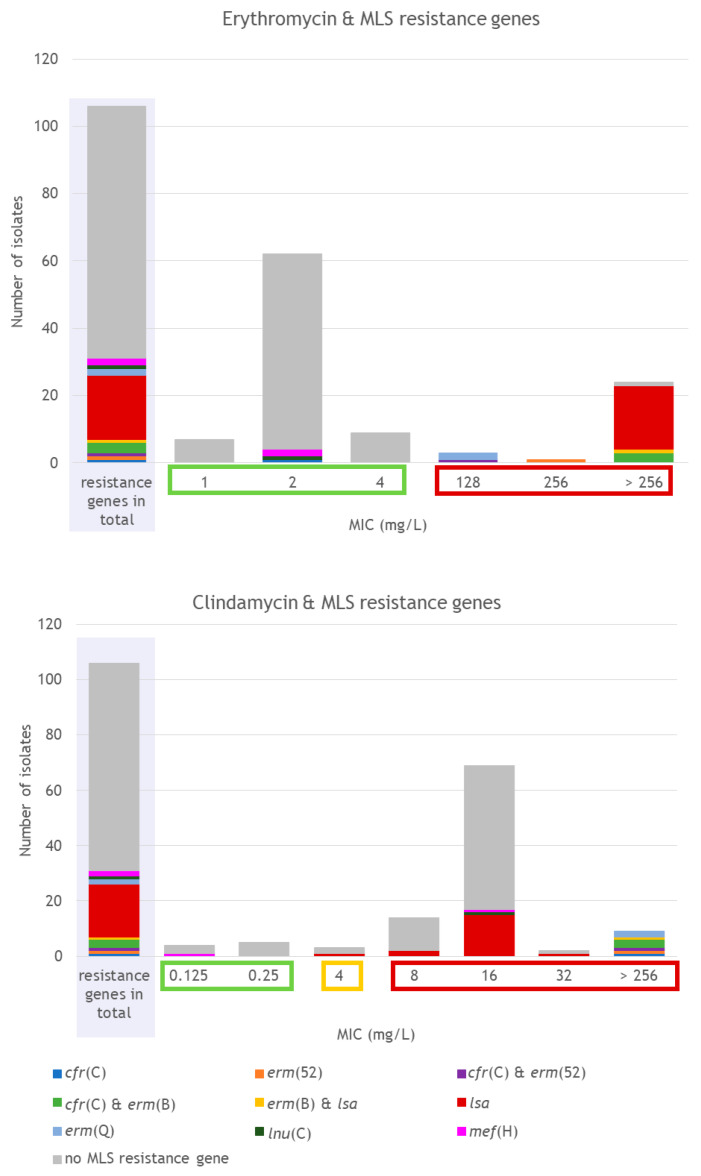
Detected MLS resistance genes and MIC values for erythromycin and clindamycin. Results for RT014/0 isolates are not included in this figure (they were excluded from the phenotypic AST due to insufficient growth on the control plates). Furthermore, the intrinsic resistance gene *cplR* is not shown in this figure. MIC values for erythromycin are marked as wild type (green) or non-wild type (red) according to the EUCAST epidemiological cut-off value for erythromycin [45]. MIC values for clindamycin are marked as susceptible (green), intermediate (yellow) or resistant (red) according to CLSI breakpoints for clindamycin [40].

Besides this intrinsic resistance gene, 28.7% (31/108) of the isolates carried AMR determinants conferring resistance to antimicrobial agents of the MLS group (Figure 6). In five of these isolates, two distinct MLS resistance genes were present.

**Figure 7:**
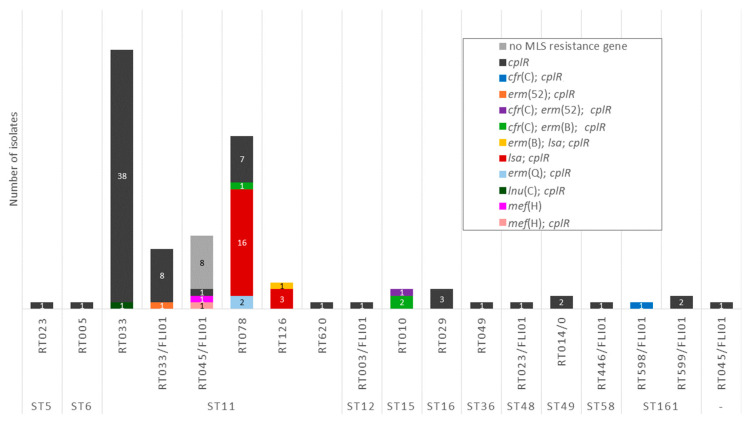
Presence of MLS resistance genes depending on ST and RT.

Several genes coding for 23S rRNA methyltransferases were detected. The *erm*(B) gene was present in four isolates belonging to ribotypes RT010 (*n* = 2), RT078 (*n* = 1), and RT126 (*n* = 1). The *erm*(Q) gene was detected in two RT078 isolates, and *erm*(52) was present in two isolates belonging to RT010 and RT033/FLI01. The *cfr*(C) gene was found in three RT010 isolates, one RT078 isolate and one RT598/FLI01 isolate. In addition, *lnu*(C), which codes for a lincosamide nucleotidyltransferase, was present in one RT033 isolate. A gene of the *lsa* family coding for an ABC-F type ribosomal protection protein was detected in 16 RT078 and four RT0126 isolates. The *mef*(H) gene was present in two isolates of RT045/FLI01 (Figure 7).

Phenotypic testing for erythromycin showed MIC values for erythromycin that ranged from 1 to >256 mg/L (Figure 6). A total of 26.4% (28/106) of the isolates were classified as non-wild type (NWT) to erythromycin (ECOFF 4 mg/L [45]). These isolates belonged to RT010 (100%, 3/3), RT033/FLI01 (11.1%, 1/9), RT078 (76.9%, 20/26), and RT126 (100%, 4/4). In 96.4% (27/28) of the erythromycin NWT isolates, other MLS resistance genes than *cplR* were detected. The NWT isolate, without an additionally detected MLS resistance determinant, belonged to RT078. Moreover, two isolates (both RT045/FLI01) with erythromycin MICs of 2 mg/L harbored the gene *mef*(H).

For clindamycin, MIC values ranged from 0.125 to >256 mg/L (Figure 6). In total, 8.5% (9/106) of the isolates were classified as susceptible, 2.8% (3/106) as intermediate, and 88.7% (94/106) as resistant. All of the susceptible isolates belonged to RT045/FLI01. In these isolates, the intrinsic resistance gene *cplR* was not detected. In all nine isolates that revealed a MIC of >256 mg/L, MLS resistance genes were detected, including all isolates with detected *erm* genes (*n* = 8) or *cfr*(C) (*n* = 5). The isolate harboring *lnu*(C) (RT033) showed a MIC of 16 mg/L for clindamycin. Isolates carrying the *lsa* gene without other MLS (except for *cplR*) resistance genes revealed MICs between 4 and 32 mg/L. The isolate harboring *mef*(H) and *cplR* showed a MIC of 16 mg/L for clindamycin; the isolate harboring solely *mef*(H) showed a clindamycin MIC of 0.125 mg/L. This observation might be explained by the fact that clindamycin is not a substrate of Mef(H) (Mef: macrolide efflux) [42]. A large proportion of isolates was classified as resistant to clindamycin, but no known resistance gene (except for *cplR*) was detected (61.3%, 65/106).

### 2.4. Metronidazole

Metronidazole exhibits potent activity against *C. difficile*, and resistance remains uncommon. However, sporadic reports of isolates with reduced susceptibility have emerged in various countries [43]. The various and presumably multifactorial resistance mechanisms for metronidazole in *C. difficile* are not yet fully understood and include alterations to metabolism (nitroreductases, DNA repair, iron uptake, and biofilm formation) [15].

In all isolates, *nimB* was present. This gene codes for a cryptic heme-dependent 5-nitroimidazole reductase [46]. However, a BLASTn search did not detect the metronidazole resistance–conferring plasmid pCD-Metro in any of the isolates [47]. Phenotypically, all isolates (106/106, 100%) were classified as susceptible with MICs of 0.125 to 0.5 mg/L (breakpoint 2 mg/L [48]). The isolates with MICs of 0.5 mg/L (15.1%, 16/106) belonged to four different RT lineages (RT023/FLI01 (*n* = 1), RT033 (*n* = 9), RT033/FLI01 (*n* = 3), and RT078 (*n* = 3)), whereas isolates showing the lowest tested MIC of 0.125 mg/L (14/106, 13.2%) belonged to ten different RT lineages (RT003/FLI01 (*n* = 1), RT005 (*n* = 1), RT023 (*n* = 1), RT029 (*n* = 2), RT033 (*n* = 1), RT033/FLI01 (*n* = 1), RT045/FLI01 (*n* = 4), RT049 (*n* = 1), RT078 (*n* = 1), RT126 (*n* = 1)).

### 2.5. Tetracycline

Tetracycline resistance in *C. difficile* varies geographically and depending on the ribotype lineage. Resistance is mainly mediated by *tet* genes encoding ribosomal protection proteins [15].

**Figure 8:**
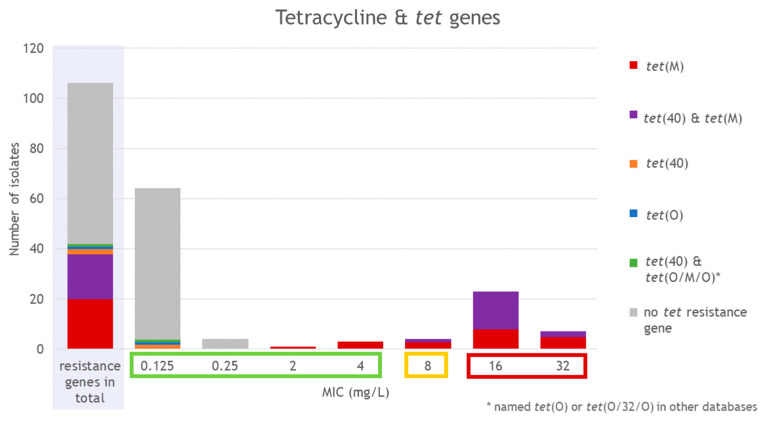
Detected tetracycline resistance genes and MIC values for tetracycline. Results for RT014/0 isolates are not included in this figure (they were excluded from the phenotypic AST due to insufficient growth on the control plates). MIC values are marked as susceptible (green), intermediate (yellow) or resistant (red) according to CLSI breakpoints [40].

**Figure 9:**
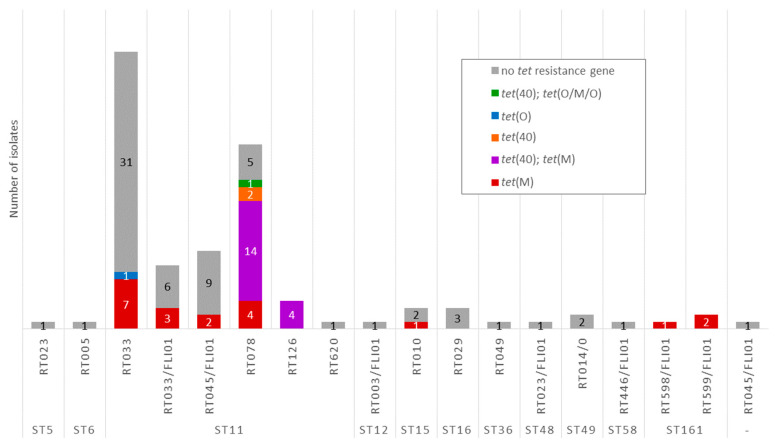
Presence of *tet* genes depending on ST and RT.

Among all investigated isolates, *tet* resistance genes were present in 38.9% (42/108) of the isolates (Figure 8). The *tet*(M) gene was present in 38 isolates, while *tet*(40) was identified in 21 isolates from two ribotypes, RT078 (*n* = 17) and RT126 (*n* = 4). In 20 of these *tet*(40)-positive isolates, the gene co-occurred with *lsa* and *aadE* genes. In 18 isolates, *tet*(M) and *tet*(40) were both present (14 RT078 and four RT126 isolates). The *tet*(O) gene was detected in one RT033 isolate. The mosaic gene *tet*(O/M/O) (also named *tet*(O/32/O) or *tet*(O) depending on the database) was present in one RT078 isolate (Figure 9).

Phenotypic testing for tetracycline revealed that most isolates showed the lowest tested MIC of ≤0.125 mg/L (60.4%, 64/106). Overall, 67.9% (72/106) of the isolates were classified as susceptible with MICs up to 4 mg/L. Four isolates had a MIC of 8 mg/L and were, therefore, classified as intermediate for tetracycline (Figure 8). The tetracycline-resistant isolates revealed MICs of 16 to 32 mg/L (28.3%, 30/106) and belonged to RT033 (15.4%, 6/39), RT033/FLI01 (22.2%, 2/9), RT045/FLI01 (16.7%, 2/12), RT078 (61.5%, 16/26), and RT126 (100%, 4/4). All isolates with a MIC ≥2 mg/L harbored *tet*(M). When isolates harbored *tet*(M) and *tet*(40), MICs of 8 to 32 mg/L were obtained. The four isolates with other *tet* resistance genes (*tet*(40), *tet*(O), or *tet*(O/M/O)) showed MICs of 0.125 mg/L (Figure 8).

### 2.6. Vancomycin

Vancomycin remains one of the primary therapeutic compounds against *C. difficile* infections in humans, and confirmed resistance in this species is rare [49]. The gene cluster *vanG* seems to be widespread in *C. difficile*, but this cluster does not seem to confer vancomycin resistance [15].

**Figure 10:**
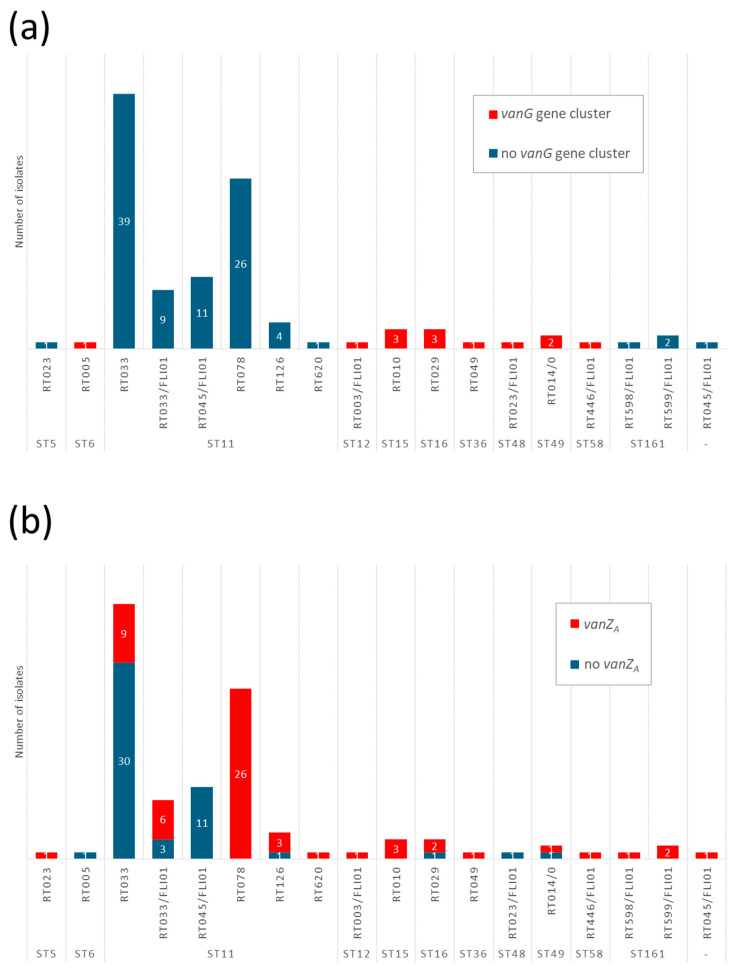
Presence of selected *van* genes depending on ST and RT. (**a**) Presence of *vanG* gene cluster depending on ST and RT; (**b**) Presence of *vanZ_A_* depending on ST and RT.

However, different *van* genes have been variably detected depending on the database used for analysis. Results of RGI of CARD showed that every isolate harbored alleles of *vanY*, *vanT* and *vanW* (Table S3.2). In 13 isolates (12.0%; 13/108), the genes *vanG* and *vanXY* were detected by this database (Table S3.2). In these 13 isolates, the *vanG* gene cluster (composed of *vanG*, *vanRC*, *vanSC* and *vanTC*) was detected with ABRicate and the database MEGARes (Table S3.6). The isolates positive for this gene cluster belonged to RT003/FLI01, RT005, RT010, RT014/0, RT023/FLI01, RT029, RT049, and RT446/FLI01 (Figure 10). Furthermore, *vanZ_A_* was detected in 54.6% (59/108) of the isolates (Table S3.6; Figure 10).

In one RT023 isolate, a *van*A-G was detected with a coverage of 87% via ABRicate and the ARG-ANNOT database (Table S3.7). With AMRfinderplus, the VanR-ABDEGLN family response regulator transcription factor *vanR* was found in two isolates (RT003/FLI01 and RT023) and *vanS-Cd*_T369I in one RT005 isolate (Table S3.1).

Phenotypic testing for vancomycin resistance revealed that all isolates were classified as susceptible, with MICs of 0.5 to 2 mg/L. Only one RT005 isolate showed a MIC of 2 mg/L.

### 2.7. Aminoglycosides

**Figure 11:**
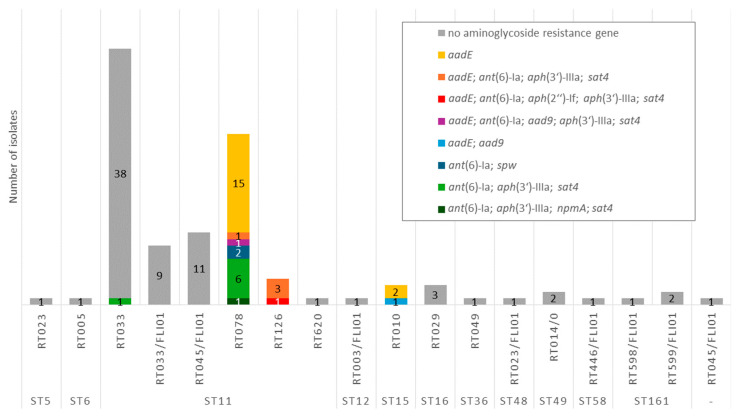
Presence of aminoglycoside resistance genes depending on ST and RT.

*C. difficile* is intrinsically resistant to aminoglycosides [15]. Genotypic analysis of the isolates showed that AMR genes associated with aminoglycoside resistance were present in 31.5% (34/108) of the investigated isolates. These isolates belonged to RT078 (100%, 26/26), RT126 (100%, 4/4), RT010 (100%, 3/3), and RT033 (2.6%, 1/39) (Figure 11). All of them carried members of the *ant*(6) gene family: *ant*(6)-Ia and/or *aadE*.

The RT033 isolate additionally harbored the streptothricin resistance gene *sat4* and the aminoglycoside resistance gene *aph*(3′)-IIIa. All RT010 isolates possessed the *aadE* gene, and one of them also harbored the *aad9* gene. The gene *spw* was present in two RT078 isolates. All RT126 isolates showed the genes *aadE*, *ant*(6)-Ia, *sat4* and *aph*(3′)-IIa; in one of these isolates, *aph*(2″)-If was also present. The gene *npmA* was detected in one RT078 isolate.

### 2.8. Further Results

All isolates contained the *cdeA* gene that codes for a multidrug efflux transporter. Mutations in *rpoB* that could confer resistance to rifamycins were not detected.

### 2.9. Summary: Agreement/Discordance Between Phenotypic and Genotypic Antimicrobial Resistances

Agreement and discordance of detected phenotypic and genotypic AMR varied among the tested antimicrobial agents (Table 1).

A high agreement (100%) could be seen for most of the tested antimicrobial agents. The lowest agreement was detected for isolates harboring amino acid substitutions in GyrB, but classified as susceptible for moxifloxacin (12.1% agreement). Another low value (28.6% agreement) was obtained for isolates classified as NWT for erythromycin. Here, the low agreement was caused by the absence of AMR determinants known to confer erythromycin-resistance.

Most frequently, the presence of the same AMR determinants (or absence of resistance determinants) resulted in comparable MIC values (seen for β-lactams, metronidazole and moxifloxacin). Regarding the results for tetracycline and *tet* genes, it could be seen that only certain genes resulted in elevated MIC values (*tet*(M) or *tet*(M) and *tet*(40)), but these MIC values covered a comparatively wide range (2 to 32 mg/L). A more diverse picture was obtained for MLS antimicrobial agents, with isolates being classified as NWT for erythromycin without a detected “classical” antimicrobial resistance gene, e.g., *erm*(B). If antimicrobial resistance genes were present, the same gene (combination) in the respective isolates resulted in the same MIC values for erythromycin. For clindamycin, a reliable agreement was seen for the isolates classified as susceptible (no *cplR* gene) and isolates with a MIC of >256 mg/L (all of these isolates harbored a resistance gene). For isolates showing MIC values in between (4 to 32 mg/L), such a concordance could not be detected. For vancomycin and *van* genes, it could be seen that the presence of the detected *van* genes did not result in phenotypic resistance; all isolates were classified as susceptible. As these *van* genes are not described in the current literature to confer resistance, they were categorized as antimicrobial resistant determinants that do not confer resistance [15].

## 3. Discussion

Various methods are available for phenotypic antibiotic resistance testing, including the agar diffusion test, agar dilution test (gold standard for *C. difficile*), epsilometer test, and broth microdilution test [53,54]. The results obtained can be used to predict whether a strain is susceptible or resistant to a particular antimicrobial agent. However, these tests can be very complex and time-consuming [55]. Furthermore, they can be very expensive depending on the method, number of isolates and tested antimicrobial agents. In contrast, whole-genome sequencing technologies are becoming increasingly faster and cheaper [56]. With these technologies, it is possible to detect antibiotic resistance determinants quickly [15]. However, the challenge is that the genotypic resistance profile does not always coincide with the phenotypic resistance profile [15], as was also demonstrated in this study. Therefore, it is essential to compare phenotypic and genotypic resistance to uncover further possible resistance mechanisms in *C. difficile*.

In this study, various phenotypic and genotypic antimicrobial resistances were identified. This wide range of identified antimicrobial resistances in *C. difficile* has already been described in other studies. In the ClosER study, in which almost 3500 strains of different RT lineages from different European countries were tested, phenotypic resistance was detected for all tested antimicrobial agents (metronidazole, vancomycin, fidaxomycin, rifampicin, moxifloxacin, clindamycin, imipenem, chloramphenicol, and tigecycline), with resistance rates ranging from 0.03% (tigecycline and fidaxomicin; 1/3499) to 51.3% (clindamycin; 1794/3498) [57]. In another study, 10,330 *C. difficile* genomes of various STs were screened for antimicrobial resistance determinants, finding various resistance determinants for the classes of fluoroquinolones, MLS antibiotics, tetracyclines, ansamycins, β-lactams, sulfonamides, aminoglycosides, as well as for vancomycin, metronidazole, linezolid, trimethoprim, and chloramphenicol [58]. The results of our study fit in well here, as various antimicrobial resistances were found in the isolates examined that belonged to different RT/ST linages, even if these originated from the same source and geographical region (i.e., bovine isolates from Germany).

The investigated isolates here largely belong to ST11 (83.3%; *n* = 90/108). This ST exhibits an extensive repertoire of antimicrobial resistances [16]. RT078, in particular, is a livestock-associated strain that is found as the dominant RT in pig and cattle herds [12]. The fact that this lineage shows resistance against various antimicrobial agents can be explained, among other things, by the worldwide use of antibiotics in livestock farming—not only for the treatment of diseases but also as growth promoters, despite this use being banned in the European Union in 2006 [14]. Furthermore, antimicrobial agents, such as, for example, streptomycin or oxytetracycline, are worldwide used for plant protection in the agricultural sector [59]. A total of 92.2% (83/90) of the investigated ST11 isolates showed phenotypic resistance or were classified as non-wild type to at least one of the antimicrobials tetracycline, moxifloxacin, erythromycin, and clindamycin (Supplementary Materials Table S1). This value is well above the phenotypic resistance rate of 48.1% for ST11 isolates to these antibiotics measured by Knight et al., who tested a total of 185 strains [16]. This discrepancy is primarily due to the different clindamycin resistance rates. In this study, the clindamycin resistance rate was 87.8% (79/90), and only nine isolates were classified as susceptible (all RT045/FLI01). In the study by Knight et al., 76.8% of the tested isolates were classified as susceptible [60]. If the clindamycin resistance results are disregarded, the phenotypic resistance rate (tetracycline, moxifloxacin, and erythromycin) decreases to 43.3% (*n* = 39/90). Antibiotic resistance rates in *C. difficile* differ not only with regard to RT and ST, but the origin (geographical and source) of the isolates also plays an important role [16]. While the strain collection in Knight et al. consists of human and non-human strains from various continents [16], the investigated isolates of this study originated from German cattle herds. According to Regulation (EU) No 37/2010 on pharmacologically active substances and their classification regarding maximum residue limits in foodstuffs of animal origin (version 15.10.2025), clindamycin is not allowed to be used in food-producing animals. However, some lincosamides, such as lincomycin and pirlimycin, are approved for cattle and calves in Germany; they may select for lincosamide resistance genes that also confer clindamycin resistance. The narrow selection of the sources examined (German cattle) may nevertheless influence the clindamycin resistance rate. One possible explanation could be a genetic relationship between the isolates. In a study investigating porcine and human RT078 strains from four European countries, it was demonstrated that 85% of the strains examined showed genetic relatedness to other strains, 103 of these 158 strains (65.2%) even formed one genetically related cluster [61]. ST11 is common in livestock [14]. It is possible that the investigated ST11 isolates are particularly well adapted to the conditions in cattle herds, also due to existing resistances. Furthermore, a common source, such as a connection between individual farms through retail chains, is conceivable.

### 3.1. Beta-Lactams and bla_CDD_ Genes

For the tested beta-lactam antimicrobial agents, the obtained agreement between phenotypic and genotypic AMR was 100% (Table 1). Chromosomal class D beta-lactamase genes, mostly *bla*_CDD-1_, were found in all isolates investigated. These genes confer intrinsic resistance to beta-lactam antibiotics (including penicillins, cephalosporins and monobactams) [15,62]. Carbapenems are no target of beta-lactamases [15]. Isolates of RT003/FLI01 (*n* = 1), RT010 (*n* = 3) and RT023/FLI01 (*n* = 1) harbored the gene *bla*_CDD-2_. Therefore, a ribotype dependency regarding the presence of *bla*_CDD-1_ or *bla*_CDD-2_ seems possible. A lineage dependency was also detected in another study [63]. In this study, the RT010 isolate possessed *bla*_CDD-2_ as well; no RT003 or ST48 (RT023/FLI01) isolate was included [63]. We measured a reduced phenotypic susceptibility to penicillin, consistent with results from other studies [64,65]. It was striking that all *C. difficile* isolates investigated in this study with MIC values of ≥2 mg/L for penicillin (which could be assigned to an ST) belonged to ST11, ST161, and ST15. All isolates with MICs of 4 mg/L belonged to ST161 (RT598/FLI01 and RT599/FLI01). Therefore, a lineage dependency regarding the MIC for penicillin might be investigated in further studies. All isolates were susceptible to the carbapenem meropenem. Similar results have been found in other studies, with only a few resistant strains [65,66,67]. Our results do not indicate that the presence of *bla*_CDD-1_ or *bla*_CDD-2_ results in lower or higher MICs for penicillin; a lineage (RT/ST) dependency regarding MIC results seems more likely. Due to the occasionally small number of isolates belonging to special RTs, a statistical analysis is not useful and should only be performed on a larger isolate collection.

### 3.2. Vancomycin and van Genes

The *vanG* gene cluster was only detected in specific ST/RT lineages; ST11, ST161, and ST5 were negative for this cluster. This was also seen in other studies, with RT078 and RT126 isolates (they belong to ST11) being negative for this cluster [68,69]. Phenotypically, all isolates tested here were susceptible to vancomycin, in agreement with the results of other studies [16,69,70]. No dependency of *vanG* gene cluster presence and MIC values for vancomycin was detected; isolates with the *vanG* gene cluster showed MIC values ranging from 0.5 to 2 mg/L for vancomycin. The prevalence of *vanZ_A_* differed between the different RT lineages. Whereas all RT078 harbored this gene, there were some lineages (e.g., RT033, RT126, RT029) with isolates positive and negative for *vanZ_A_*.

### 3.3. Tetracycline and tet Genes

All isolates that harbored at least one *tet* resistance gene belonged to ST11, ST161, or ST15. Of the 26 RT078 isolates examined, 18 possessed a *tet*(M) gene (69.2%). A similar percentage (76.1%, 83/109) was determined in a phylogenetic analysis of clinical Scottish RT078 genomes [52]. This indicates that RT078 strains exhibit similar rates of antimicrobial resistance determinants, regardless of their source. A discrepancy between the phenotype and genotype resistance type was observed in the results for tetracycline. Not all isolates harboring *tet* genes were classified as resistant. All isolates tested here with a tetracycline MIC of at least 2 mg/L harbored the resistance gene *tet*(M); if both *tet*(M) and *tet*(40) were detected, the isolates exhibited a tetracycline MIC of at least 8 mg/L. *tet*(40), *tet*(O) or the combination of *tet*(40) and *tet*(O/M/O) alone without *tet*(M) did not cause increased MIC values against tetracycline; all isolates with these genes (*n* = 4) showed a MIC value of 0.125 mg/L. A link between *tet*(M) and tetracycline resistance has already been detected in other studies. For example, in a study by Bakker et al., all RT078 isolates tested with *tet*(M) had a MIC of at least 8 mg/L [61]. In a Chinese study, all tetracycline-resistant isolates harbored *tet*(M), but there were also *tet*(M)-positive isolates that were susceptible to tetracycline [71]. Specific MIC values were not specified; the CLSI breakpoint for tetracycline was used [71]. Among the *C. difficile* isolates tested here, there were also *tet*(M)-positive isolates that were classified as “susceptible” to tetracycline. One possible conclusion could be that the presence of *tet*(M) leads to increased MIC values (≥2 mg/L), which, however, do not always necessarily result in a classification as resistant (≥16 mg/L). This could be due to different alleles of the *tet*(M) gene, which could lead to different MIC values (Table S3.5). An increase in the MIC value could be enhanced by the additional presence of the efflux pump gene *tet*(40) (≥8 mg/L). Considering these results, the relevance of the breakpoint used and the necessity of specifying the measured MIC values become apparent in order to be able to make statements about the relationship between phenotypic and genotypic antimicrobial resistance.

### 3.4. Erythromycin, Clindamycin and MLS Resistance Genes

As described for the *tet* resistance genes, macrolide, lincosamide, and streptogramin resistance genes were present only in ST11, ST161, and ST15. The fact that ST11, in particular, represents a lineage with a broad antibiotic resistance repertoire has already been described in other publications [16]. Isolates with *erm* genes showed MICs of at least 128 mg/L for erythromycin and >256 mg/L for clindamycin. The gene *cfr*(C) was detected in five isolates, in four of them together with *erm* genes. The isolate harboring solely *cfr*(C) (and *cplR*) was classified as resistant to clindamycin (>256 mg/L) and as wild type (WT) for erythromycin (2 mg/L). A *lsa-*like gene was present in RT078 (16/26; 61.5%) and RT126 (4/4; 100%) isolates. Phenotypically, all these isolates had an erythromycin MIC of >256 mg/L, with only one isolate possessing an additional *erm*(B) gene. This resulted in a comparably low calculated agreement of phenotypic and genotypic AMR (28.6%, Table 1). Erythromycin-resistant *C. difficile* strains lacking *erm* genes have been frequently described [15]. Whether this *lsa* gene is somehow related to erythromycin resistance, even though known *lsa* genes do not confer macrolide resistance, needs further investigation. Possibly, it is part of a genetic element that contains more unknown resistance genes that were not detected in our analysis. The intrinsic pleuromutilin, lincosamide and streptogramin A resistance gene *cplR* was present in all isolates except for nine RT045/FLI01 isolates; a lineage dependency seems likely. All isolates lacking *cplR* were classified as susceptible to clindamycin; isolates with *cplR* were classified as intermediate or resistant to clindamycin. For this gene, a high agreement of phenotypic and genotypic resistance can be stated.

### 3.5. Fluoroquinolones and Substitutions in Gyrase Proteins

All ST11 and ST161 isolates tested harbored the amino acid substitutions S366V and S416A in GyrB. Isolates with these two substitutions showed a maximum MIC of 4 mg/L for moxifloxacin. The calculated agreement between phenotypic and genotypic AMR was low (12.1%) for moxifloxacin-susceptible isolates, as many isolates harbored these amino acid substitutions (Table 1). However, it is known that different substitutions confer different levels of resistance [15], a fact that could explain the results achieved in this study. Isolates with the additional substitution D426N showed MIC values of 8 mg/L, and isolates with the substitution T82I in GyrA showed MIC values of 16 mg/L. These results are in line with other studies, as T82I is associated with high-level resistance, whereas other amino acid substitutions lead to lower MICs [15]. Overall, 5.6% (5/90) of the ST11 isolates were resistant to moxifloxacin (MIC ≥ 8 mg/L). This value is far below the result of an ST11 study, in which a resistance rate of 25.9% was measured, with the results strongly dependent on the respective RT; RT126 had the highest resistance rate of 49.3% and RT033/288 the lowest resistance rate (3.0%) [16]. In this study by Knight et al., human and non-human strains from various continents were tested [16]. Our cattle isolate collection consists largely of RT033 isolates (*n* = 48), which will have significantly influenced the result, as well as the narrow selection of the sources examined (German cattle) as described above.

### 3.6. Aminoglycoside Resistance Genes

Genes of the aminoglycoside resistance gene family *ant*(6) (*ant*(6)-Ia and *aadE*) were found, alone or in combination with other aminoglycoside resistance genes, in all RT078, RT126 and RT010 genomes examined. In another study, it was detected that aminoglycoside resistance gene clusters in ST11 showed high similarity to clusters in *Erysipelothrix rhusiopathiae* and could have been transferred to *C. difficile* by horizontal gene transfer in the gastrointestinal tract of farm animals—this would explain the clustering in the livestock-associated strains (including RT078) [16]. This assumption is reinforced by the fact that some of the aminoglycoside resistance genes have already been described in connection with mobile genetic elements [72].

### 3.7. Summary

For most of the compared phenotypic and genotypic AMR, 100% agreement can be stated. Still, there are some combinations of presence/absence of AMR determinants and MIC results that need further investigation. The lowest agreements were achieved for moxifloxacin and isolates harboring mutations that resulted in amino acid substitutions (here, it could be seen that different substitutions caused different MIC levels), and for erythromycin-resistant isolates without a known AMR determinant present. Furthermore, detected *tet* genes caused comparably wide MIC ranges and, therefore, isolates had to be classified differently (susceptible to resistant), even though they harbored the same AMR determinants. This shows the need for further investigations in this field, and the need to elucidate genetic AMR mechanisms and their consequences for phenotypic AMR results. Furthermore, the obtained results underline the importance of transparent reporting of obtained MIC values and used breakpoints in studies instead of just categorizing isolates as resistant or susceptible, as concrete MIC values can represent a significant added value for interpretation.

### 3.8. Study Limitations

To our knowledge, this is the first study combining phenotypic and genotypic AMR characterization of bovine *C. difficile* isolates from Germany and provides valuable insight into the AMR characteristics of these isolates. However, the present study has some limitations. With collection dates between 2010 and 2012, the isolate collection does not necessarily reflect the current situation of circulating *C. difficile* strains in German cattle husbandry. Furthermore, the isolates originated from only six German federal states, so there are geographical limitations. Most of the isolates belonged to ST11 (90/108), which reflects the dominance of this lineage in the sampled German cattle farms, but complicates a comparison of detected AMR between different lineages. Due to this imbalance, a statistical analysis was not expedient and only a descriptive analysis was performed. Furthermore, no data concerning farm-level antimicrobial use were available.

## 4. Materials and Methods

### 4.1. Isolate Collection

We selected 108 bovine *C. difficile* isolates from our *C. difficile* isolate collection. These strains had originally been recovered from fecal samples or rectal swabs of diarrheic calves collected between January 2010 and August 2012 within a multicentre sampling effort involving eight federal laboratories from six German federal states as previously described [36]. The isolates are stored in a −80 °C freezer in Cryobank^®^-tubes (Mast Diagnostica, Reinfeld, Germany). To get comprehensive insights into the antimicrobial resistance characteristics of *C. difficile* in German bovine isolates, we included isolates that originated from different farms. The isolates belonged to different ribotypes and sequence types (Table 2). The origin (Federal State) of each isolate is listed in Table S4.

### 4.2. Whole Genome Sequencing of the Clostridioides Difficile Isolates from Cattle

To investigate the genomic antimicrobial resistance (AMR) determinants of the isolates, DNA extraction was performed with the DNeasy Blood and Tissue kit (Qiagen, Hilden, Germany). Therefore, isolates were cultured for 24 to 48 h (37 °C, anaerobic conditions) on blood agar plates previously. Whole genome sequencing was performed with the Illumina^®^ Miseq™ platform.

The raw sequencing reads were analyzed with the Clostyper workflow version 0.5.0. (https://gitlab.com/FLI_Bioinfo/clostyper, (accessed on 17 April 2026). Briefly, the raw sequencing reads were quality-checked using fastp v0.23.2, which reports the total number of sequenced bases and reads, GC content, Phred quality scores, and duplicated sequence regions. Sequencing depth was inferred from the ratio of total sequenced bases to the reference genome size (4.1 Mb). Raw reads were taxonomically classified with Kraken2 v2.1.2, and intra- and cross-species contamination was assessed with ConFindr v0.7.4. Genome assemblies were generated within Clostyper using the assembler SKESA 2.4.0 and annotated with Bakta v1.9.3 in default mode. Assembly statistics were calculated with SeqKit v2.8.2, including the number of contigs, total length, N50, GC content, and ambiguous bases. For assembled genomes, taxonomic classification was again performed with Kraken2 v2.1.3, while CheckM v1.2.3 was used to evaluate completeness, contamination, and heterogeneity. Closely related reference genomes were identified using ReferenceSeeker v1.8.0, and average nucleotide identity (ANI) was estimated with FastANI v1.34 relative to *C. difficile* CD196. Based on cgMLST, a cluster tree was constructed (Figure S1).

### 4.3. Investigation of Antimicrobial Resistance Determinants

AMR genes were identified using ABRicate (v1.0.1) and AMRFinderPlus (v3.10.42) implemented in the Clostyper pipeline with default parameters. Searches with ABRicate (version 1.0.1) were conducted against multiple curated databases, including CARD, NCBI, ResFinder, MEGARes, and ARG-ANNOT (updated 10 January 2023). Hits were considered valid with a minimum sequence identity of 80% and a minimum coverage of 80%. In addition, AMRFinderPlus (version 3.10.42) with its database (version 2022-12-19.1) was used to identify antimicrobial resistance genes. All genomes were further screened for resistance-associated point mutations in *gyrA*, *gyrB* and *rpoB* genes.

### 4.4. Antimicrobial Susceptibility Testing

Antimicrobial susceptibility testing was performed by the agar dilution procedure according to CLSI document M11 [73]. Therefore, antimicrobial agent-containing plates (Brucella agar (Carl Roth^®^, Karlsruhe, Germany) supplemented with 5 µg/mL hemin (Carl Roth^®^, Karlsruhe, Germany), 1 µg/mL vitamin K1 (Carl Roth^®^, Karlsruhe, Germany) and 5% defibrinated sheep blood (Xebios Diagnostics, Düsseldorf, Germany)) with concentrations from 0.125 to 256 mg/L (twofold serial dilution) according to CLSI document M11 were prepared [73]. As quality control, the reference strains *Clostridioides* (*Clostridium*) *difficile* ATCC^®^ 700057, *Bacterioides* (*B.*) *fragilis* ATCC^®^ 25285, and *B. thetaiotaomicron* ATCC^®^ 29741 were included. After the initial culture from the preserved Cryobank^®^ tubes and subculturing, isolates were precultured for 24 to 48 h for inocula preparation. All at 37 °C, anaerobic conditions on brucella agar plates with hemin, vitamin K_1_ and 5% defibrinated sheep blood. The density of the inocula was adjusted from 0.5 to 0.9–1.1 McFarland to obtain sufficient growth and evaluability of the stamped bacterial suspensions. With a replicator, inocula were stamped on the antimicrobial agent-containing plates. These plates were incubated for 42 to 48 h (37 °C, anaerobic conditions; aerobic conditions for the respective control plates). MIC values were determined for penicillin (Carl Roth^®^, Karlsruhe, Germany), meropenem (Supelco^®^, Sigma-Aldrich RTC, Laramie, WY, USA), tetracycline (Carl Roth^®^, Karlsruhe, Germany), moxifloxacin (Supelco^®^, Sigma-Aldrich RTC, Laramie, WY, USA), vancomycin (Carl Roth^®^, Karlsruhe, Germany), metronidazole (Sigma-Aldrich^®^, Saint Louis, MI, USA), erythromycin (Carl Roth^®^, Karlsruhe, Germany) and clindamycin (Supelco^®^, Sigma-Aldrich RTC, Laramie, WY, USA). Therefore, the lowest concentration that completely inhibited visible growth was determined for each isolate–antimicrobial agent combination. Breakpoints according to CLSI were used for penicillin, meropenem, tetracycline, moxifloxacin and clindamycin [40]. For vancomycin and metronidazole, breakpoints according to EUCAST were used [48]. The EUCAST epidemiological cut off values were used for erythromycin [45].

### 4.5. Agreement Between Phenotypic and Genotypic Antimicrobial Resistance

AMR genotype–phenotype concordance was assessed based on the following assumptions: genotypic AMR determinants known or proposed to confer resistance to the respective antimicrobial agent were expected to result in elevated MIC values and classification as intermediate, resistant, or non-wild type; absence of relevant AMR determinants was expected to result in non-elevated MIC values and classification as susceptible or wild type; AMR determinants not associated with resistance to the respective antimicrobial agent were considered non-resistance-conferring; and determinants with insufficient evidence for a resistance-conferring effect were also classified as non-resistance-conferring.

## Figures and Tables

**Table 1:** Agreement between phenotypic and genotypic antimicrobial resistance results.

	Phenotypic Classification	Detected Antimicrobial Resistance Determinants (ARDs)	Number of Isolates			Agreement Between Phenotypic and Genotypic AMR
			with ARDs That Confer Resistance	with ARDs That Do Not Confer Resistance	Without ARDs	
PEN	S	-	-	-	-	-
	I	*bla*_AHM_; ***bla*****_CDD-1_**; ***bla*_CDD-2_**	35	-	-	100%
	R	*bla*_AHM_; ***bla*****_CDD-1_**; ***bla*_CDD-2_**	71	-	-	100%
MEP	S	*bla*_AHM_; *bla*_CDD-1_; *bla*_CDD-2_	-	106		100%
	I	-	-	-	-	-
	R	-	-	-	-	-
MOX	S	V130I (GyrB); **S366V (GyrB)**; **S416A (GyrB)**	87	1	11	12.1%
	I	**S366V (GyrB)**; **S416A (GyrB)**	2	-	-	100%
	R	**S366V (GyrB)**; **S416A (GyrB)**; **D426N (GyrB)**; **T82I (GyrA)**	5	-	-	100%
ERY	WT	*cfr*(C); *cplR*; *lnu*(C); ***mef*(H)**	2	68	8	97.4%
	NWT	*cfr*(C); *cplR*; ***erm*****(52)**; ***erm*(B)**; ***erm*(Q)**; *lsa*	8	20	-	28.6%
CLI	S	*mef*(H)	-	1	8	100%
	I	***cplR***; ***lsa***	3	-	-	100%
	R	***cfr*(C)**; ***cplR***; ***erm*(52)**; ***erm*(B)**; ***erm*(Q)**; ***lnu*(C)**; ***lsa***; *mef*(H)	94	-	-	100%
MEZ	S	*nimB*	-	106	-	100%
	R	-	-	-	-	-
TET	S	***tet*(40)**; ***tet*(M)**; ***tet*(O)**; ***tet*(O/M/O)**	8	-	64	88.9%
	I	***tet*(40)**; ***tet*(M)**	4	-	-	100%
	R	***tet*(40)**; ***tet*(M)**	30	-	-	100%
VAN	S	*vanA*-*G*; *vanG* gene cluster; *vanR*; *vanS*-Cd_T369I; *vanT*; *vanW*; *vanY*; *vanZ_A_*	-	106	-	100%
	R	-	-	-	-	-

Blue: expected result; Genes shown in **bold** are known or suspected to confer resistance or elevated MIC values for the corresponding antimicrobial agent [15,42,44,46,50,51,52]. PEN: penicillin, MEP: meropenem, MOX: moxifloxacin, ERY: erythromycin, CLI: clindamycin, MEZ: metronidazole, TET: tetracycline, VAN: vancomycin; S: susceptible, I: intermediate, R: resistant, WT: wild type, and NWT: non-wild type [40,45,48]; ARD: antimicrobial resistance determinant.

**Table 2:** Sequence types, ribotypes and origins of the investigated isolates.

Sequence Type	Ribotype	Number of Isolates	Origin (Federal State)
ST5	RT023	1	Baden–Württemberg
ST6	RT005	1	Thuringia
ST11	RT033	39	Baden–Württemberg (32); Lower Saxony (2); Mecklenburg–Western Pomerania (5)
	RT033/FLI01	9	Baden–Württemberg (5); Mecklenburg–Western Pomerania (4)
	RT045/FLI01	11	Baden–Württemberg (4); Lower Saxony (1); Mecklenburg–Western Pomerania (5); Schleswig–Holstein (1)
	RT078	26	Baden–Württemberg (21); Lower Saxony (1); Mecklenburg–Western Pomerania (4)
	RT126	4	Baden–Württemberg (3); Lower Saxony (1)
	RT620	1	Mecklenburg–Western Pomerania
ST12	RT003/FLI01	1	Baden–Württemberg
ST15	RT010	3	Baden–Württemberg (1); Mecklenburg–Western Pomerania (2)
ST16	RT029	3	Baden–Württemberg
ST36	RT049	1	Saxony
ST48	RT023/FLI01	1	Baden–Württemberg
ST49	RT014/0	2	Baden–Württemberg (1); Saxony (1)
ST58	RT446/FLI01	1	Baden–Württemberg
ST161	RT598/FLI01	1	Baden–Württemberg
	RT599/FLI01	2	Baden–Württemberg
- ^1^	RT045/FLI01	1	Baden–Württemberg

^1^ For this isolate, no ST could be assigned.

## Data Availability

All genome sequence data used in this study have been deposited at the National Center for Biotechnology Information (NCBI) under the BioProject accession number PRJNA1437242.

## Supplementary Materials

The following supporting information can be downloaded at: https://www.mdpi.com/article/10.3390/antibiotics15050495/s1, Figure S1: cgMLST GrapeTree; Table S1: Minimum inhibitory concentrations in mg/L; Table S2: Overview of detected antimicrobial resistance determinants; Table S3.1: Results AMRFinderPlus; Table S3.2: Results RGI + CARD; Table S3.3: Results ABRicate + NCBI; Table S3.4: Results ABRicate + CARD; Table S3.5: Results ABRicate + ResFinder; Table S3.6: Results ABRicate + megares; Table S3.7: Results ABRicate + argannot; Table S4: Origin of the isolates (Federal State).

## Abbreviations

The following abbreviations are used in this manuscript:

AMR	antimicrobial resistance
AST	antimicrobial susceptibility testing
*C. difficile*	*Clostridioides difficile*
CLSI	Clinical and Laboratory Standards Institute
ECOFF	epidemiological cut off value
EUCAST	European Committee on Antimicrobial Susceptibility Testing
MIC	minimal inhibitory concentration
MLS	macrolide–lincosamide–streptogramin
NWT	non-wild type
QRDR	quinolone resistance-determining regions
RT	ribotype
ST	sequence type
U.S.	United States of America
WT	wild type
